# Quality Assurance of the ‘MRCPsych Course’ in Wales

**DOI:** 10.1192/bjo.2022.117

**Published:** 2022-06-20

**Authors:** Megan Davies-Kabir, Ann Collins, Catherine Walton

**Affiliations:** 1 Cwm Taf Morgannwg University Health board, Tonteg, United Kingdom.; 2 Aneurin Bevan University Health Board, Caerleon, United Kingdom; 3 Cardiff and Vale University Health Board, Cardiff, United Kingdom

## Abstract

**Aims:**

The ‘MRCPsych Course’ (Membership of the Royal College of Psychiatrists) is provided to all core trainees in psychiatry in Wales by the School of Psychiatry, Health Education and Improvement Wales (HEIW), now delivered online since the start of the COVID-19 pandemic. The aims of the HEIW MRCPsych course are: to prepare core trainees for the MRCPsych exams and to set a ‘robust platform’ for speciality training at the higher level in psychiatry. We undertook a quality assurance of the 2020/21 academic year to see how content and delivery of the course were serving these aims and make recommendations for improvement.

**Methods:**

Over the course of one academic year we triangulated trainee feedback, lecturer feedback and peer review. Trainee and trainer feedback forms were sent out following every session. We developed standards and criteria for peer review and reviewed 10% of sessions. We conducted focus groups with trainees using mentimeter to structure a real time, anonymous interaction with parallel verbal and written discussions using a virtual meeting.

**Results:**

Trainee feedback forms were received for 31 lectures from an average of 11 trainees per session. 14 Lecturer feedback forms were received, and 48 trainees attended the two focus groups. 15 hours of teaching underwent peer review.

**Conclusion:**

Lecture content was universally accurate and up to date and all teachers were fluent and engaging, with almost all incorporating research data, guidelines and inspiration for further learning.

Several lecturer feedback forms requested a curriculum be provided. Some trainees requested a more exam focused approach with more MCQs.

Trainees found online sessions more accessible and convenient. The major downside being that they no longer get to know each other and feel very anonymous, which makes peer support and interaction more difficult.

Interactive engagement was the lowest scored domain overall. Interaction seemed to work best when done as a continuous process from the start and when a variety of techniques were engaged. Trainee's suggestions for increasing interactivity included quizzes, polls, breakout rooms, use of interactive tools, and a general encouragement of cameras and microphones on and active discussion throughout the session.

Speakers had no problems using the technology to deliver an online session, this triangulated with their high confidence and high satisfaction reported by lectures with HEIW practical support. Trainees reported a high satisfaction with the quality of teaching on the course.

Our conclusions have informed changes which are currently being implemented and tested.

